# Tumor control with PD-1 inhibition in a patient with concurrent metastatic melanoma and renal cell carcinoma

**DOI:** 10.1186/s40425-016-0129-x

**Published:** 2016-04-19

**Authors:** Melina E. Marmarelis, Meredith R. Davis, Nilay S. Sethi, Katherine M. Krajewksi, Rana R. McKay, Toni K. Choueiri, Patrick A. Ott

**Affiliations:** Department of Medical Oncology, Dana Farber Cancer Institute, Brigham and Women’s Hospital, and Harvard Medical School, Boston, MA USA; Department of Imaging, Dana Farber Cancer Institute, Brigham and Women’s Hospital, and Harvard Medical School, Boston, MA USA

**Keywords:** PD-1, Immune checkpoint blockade, Antibody therapy, Melanoma, Renal cell cancer, Immunotherapy, Concurrent cancer

## Abstract

Blockade of the immunological checkpoint programmed death 1 (PD-1) using monoclonal antibodies has shown robust anti-tumor activity across a broad range of solid and hematological malignancies including melanoma and renal cell carcinoma (RCC). Characteristic markers such as the presence of tumor infiltrating lymphocytes, PD-L1 status, and mutational load may be equally or even more important in predicting clinical benefit from PD-1 pathway blockade than tumor histology. This case of a patient with concurrent metastatic melanoma and metastatic RCC, both of which were controlled for more than a year after a single dose of the anti-PD-1 antibody pembrolizumab, illustrates the potential to simultaneously treat distinct immunogenic tumors with anti-PD-1 agents.

## Background

Immune checkpoint blockade using monoclonal antibodies directed against negative regulators such as cytotoxic lymphocyte antigen-4 (CTLA-4) and programmed death 1 (PD-1)/programmed death ligand 1 (PD-L1) has emerged as a powerful strategy in the treatment of different cancer types [1–3]. Both CTLA-4 and PD-1 are cell surface receptors that negatively regulate the immune response and their blockade can induce or enhance anti-tumor T cell activity. The anti-CTLA-4 monoclonal antibody ipilimumab demonstrated a survival benefit in Phase III studies for the first time in patients with advanced melanoma [2, 4], leading to approval in several countries. Durable tumor responses in patients with advanced melanoma being treated with ipilimumab yielded a plateau in the survival curve at 21 % 3 years out from study initiation [5]. Inhibition of the PD-1/PD-L1 pathway showed objective response rates of up to 40 % and superior overall survival when compared to ipilimumab in advanced melanoma [6]. Strikingly, as opposed to the relatively modest anti-tumor activity of ipilimumab outside of melanoma, PD-1 pathway inhibition is efficacious against a wide spectrum of solid and hematological malignancies including RCC, non-small cell lung cancer, bladder cancer, and Hodgkin’s lymphoma. There is evidence that tumor characteristics such as the presence of an immune cell infiltrate, expression of PD-L1 on tumor and/or immune cells, and an elevated mutational load with corresponding expression of neoantigens are predictive of anti-tumor activity with PD-1 pathway inhibition [7–10]. The broad anti-tumor activity of PD-1 pathway blockade suggests that it may be effective against different tumors present in one individual. These considerations may be critical in designing a treatment plan for a patient with metastases from different primary tumors, which poses a particular challenge in current cancer therapeutics. Here, we present a patient with concurrent metatstatic melanoma and RCC who achieved disease control of both malignancies after a single dose of the anti-PD-1 monoclonal antibody pembrolizumab.

## Case presentation

**Fig. 1:**
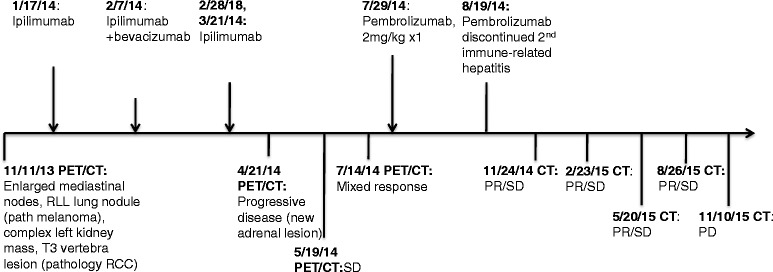
Timeline of events and treatment. CT = computerized tomography. PET = positron emission tomography. RLL = right lower lobe. RCC = renal cell carcinoma

**Fig. 2:**
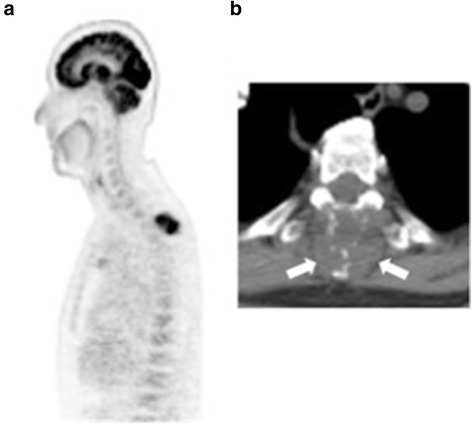
**a** PET CT demonstrating FDG uptake in an enlarged mediastinal lymph nodes, small right lower lobe lung nodule, exophytic heterogenous mass in left kidney and a 3.5 × 3.2 cm vertebral mass at T3 with SUV of 11.4. **b** Enhanced CT view of the T3 vertebral mass

**Fig. 3:**
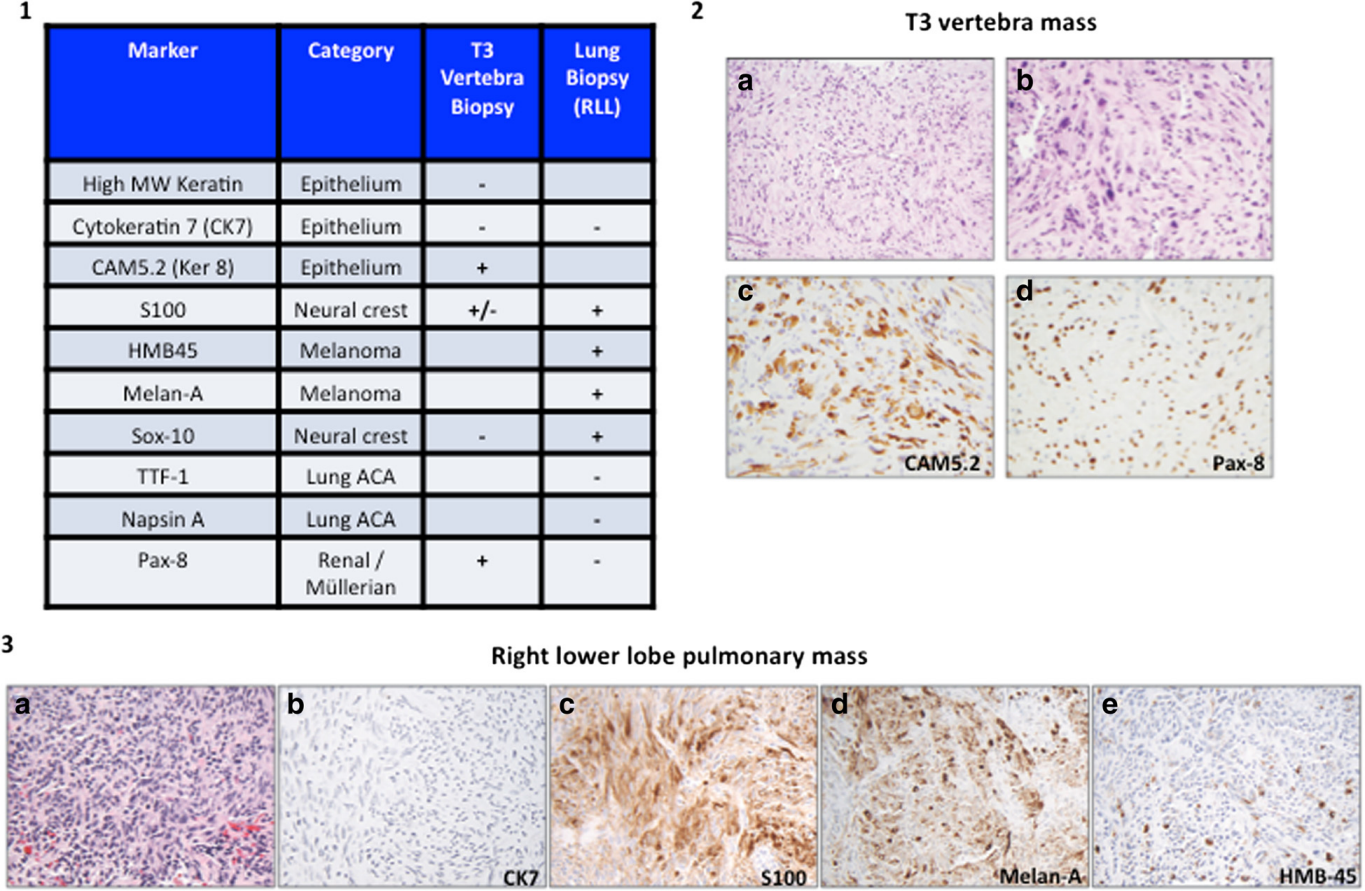
Pathology of T3 vertebral mass and right lower lobe lung lesions. Pathology of the T3 vertebral mass biopsy represents a metastasis from renal cell carcinoma, the lung lesion is consistent with a metastasis from melanoma. (1) Immunohistochemistry markers of the T3 vertebral mass and the right lower lobe (RLL) lung nodule. (2) T3 vertebral lesion: a. low power b. high power c. CAM5.2 d. Pax-8 (3) RLL lesion: a. high power b. Cytokeratin (CK7) c. S100 d. Melan-A e. HMB45

A 73-year-old man was diagnosed with T1a melanoma arising from the right shoulder in 2009. He underwent a wide excision and sentinel lymph node biopsy. Pathology review revealed a 1.64 mm melanoma, anatomic level deep III/early IV, no ulceration, 1 mitosis/mm2. Four right axillary sentinel lymph nodes were negative for involvement with melanoma. In September 2013, after experiencing hematuria, the patient underwent a cystoscopy followed by transurethral resection of a bladder tumor (TURBT), which revealed a low-grade urothelial carcinoma with no evidence of bladder invasion. He is a lifelong non-smoker. A staging computerized tomography (CT) scan revealed two right lower lobe lung nodules (2.7 cm and <1 cm), and a 6.3 cm tumor in the left kidney. A positron emission tomography computerized tomography (PET/CT) in November 2013 (Fig. 1) showed enlarged mediastinal lymph nodes in addition to FDG uptake in the lung nodules and a complex left kidney mass. A mass in the thoracic spine (T3 vertebra) and a small focus of uptake in the right sacral ala were also noted (Fig. 2). A biopsy of the T3 vertebral lesion was performed and pathologic review demonstrated RCC. A core needle biopsy of one of the right lower lobe lung nodules was also performed and unexpectedly revealed recurrent metastatic melanoma (Fig. 3). In December 2013, a brain MRI showed a subcentimeter left temporal metastasis. The patient received radiation to the T3 vertebral metastasis and stereotactic radiosurgery to the brain metastasis.

While weighing the treatment options for these two cancers, although our initial thoughts were to focus on the more aggressive melanoma (BRAF and NRAS wild type), we were motivated to design a therapy regimen that could yield efficacious responses against both cancers. Bevacizumab, the anti-Vascular Endothelial Derived Growth Factor (VEGF) directed monoclonal antibody approved for use in renal cell carcinoma, has some efficacy in advanced melanoma and was found to be safe and potentially synergistic with ipilimumab [11, 12]. The urothelial cancer was not addressed therapeutically because of the metastatic melanoma and RCC taking precedence. Based on this data, the patient received one cycle of ipilimumab at the standard dose of 3 mg/kg. Following insurance approval, he then received a cycle of ipilimumab in combination with bevacizumab 15 mg/kg every 3 weeks. Shortly after receiving the combination, the patient presented with a left sided headache, blurred vision in the left eye, and left eyelid ptosis. A brain MRI revealed a subacute hemorrhage at the site of the previously irradiated left temporal metastasis. A temporal artery biopsy was negative for temporal arteritis. The remainder of ipilimumab therapy (3rd and 4th dose) was administered without concurrent Bevacizumab given the concern that VEGF inhibition may have possibly contributed to the brain hemorrhage. The ipilimumab treatment course was also complicated by immune-related hypophysitis resulting in adrenal insufficiency, which was successfully treated with hydrocortisone.

Serial PET/CTs showed progressive disease in April 2014. Repeat PET/CT performed in July of 2014 once again revealed disease evolution with increased FDG avidity of a dominant right lower lobe lung mass, increased size and avidity of a right adrenal lesion, and increased FDG avidity of the left sided renal mass. Based on these results and the availability on an Expanded Access Program, treatment with the anti-PD-1 antibody pembrolizumab at 2 mg/kg every 3 weeks was initiated in July of 2014. After one dose, the patient developed grade 3 transaminitis (ALT > 7x ULN, AST >11x ULN, total bilirubin normal), prompting discontinuation of treatment due to presumed immune-related hepatitis. Intravenous solumedrol was administered for the treatment of immune-related hepatitis. A liver biopsy performed six days into the steroid taper (prednisone 80 mg BID for the six days prior to biopsy) showed pathological features compatible with but not diagnostic of immune-related hepatitis. Liver function tests normalized fifteen days after the initial diagnosis and remained within reference range after completion of a four-week prednisone taper.

**Fig. 4:**
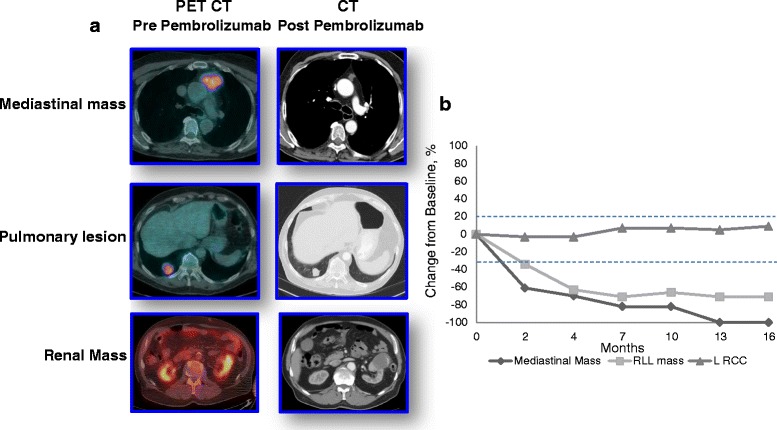
**a** PET CT images of the mediastinal mass, pulmonary lesion and renal mass. Left column: Pre- pembrolizumab; right column: 8 weeks after administration of one dose of pembrolizumab. There is near complete involution of the anterior mediastinal mass and a decrease in size of the pulmonary lesion. The size of the renal mass is unchanged. **b** Size (cm) on axial imaging (CT or PET/CT) of renal, mediastinal and dominant right pulmonary nodule over time. The last scan showed two new subcentimter pulmonary metastases and increased size of a soft tissue component of the left renal mass

Restaging CTs of the chest, abdomen, and pelvis performed in September 2014 demonstrated dramatic reduction in size of several lung metastases, while others remained stable. The right adrenal lesion, several mesenteric nodules, and a subcutaneous nodule on the left anterior abdominal wall were all substantially smaller in size. The left renal mass remained stable. Serial CTs of the chest, abdomen, and pelvis in November 2014, February 2015, May 2015, and August 2014 showed continued decrease in tumor size consistent with a partial response to treatment for a duration of 14 months (Fig. 4). Most recently, a chest, abdomen, and pelvic CT in November of 2015 showed two new subcentimeter pulmonary lesions and a satellite nodule in the primary renal cell cancer. The patient is currently undergoing re-induction therapy with pembrolizumab.

## Conclusion

We describe a patient with concurrent biopsy-proven metastatic melanoma and RCC who was treated with one dose of pembrolizumab that was complicated by immune-related hepatitis but nevertheless lead to a durable tumor response in both melanoma and RCC.

While the presence of two distinct metastatic processes was established by biopsy (melanoma in the lung and RCC in the spine at level T3), the contribution of each tumor to the overall metastatic burden cannot be determined. However, since at least one (and likely more) lung lesion(s) represent melanoma and the left renal lesion represent RCC by imaging criteria, ongoing disease control of both cancers was evident on serial imaging. It is notable that the patient developed immune-related hepatitis after only one dose of pembrolizumab. Immune-related toxicity may be associated with a favorable outcome in patients treated with CTLA-4 and/or PD-1 inhibition [13].

Multiple primary cancers, which is defined as the development of another primary cancer after diagnosis of an initial one (index cancer) in a given individual, is a relatively common occurrence. Interestingly, urinary bladder cancer is the most frequent index cancer in patients with multiple primary cancers (18 %), whereas 10 % of patients with RCC and melanoma, respectively develop a second primary [14]. No reliable data are available, to our knowledge, on frequencies of multiple primaries that have metastasized concurrently such as in our patient. It is interesting in this context that the patient’s diagnosis of a localized low grade urothelial carcinoma of the bladder immediately preceded the patient’s diagnosis of metastatic melanoma and RCC. While the non-muscle invasive bladder cancer lead to the diagnosis of melanoma and RCC in our patient, it appears unlikely that the bladder cancer contributed to the metastatic disease burden in this patient. Since bladder cancer has also been found to be responsive to PD-1 pathway inhibition, the possibility that a third solid tumor may also have been controlled by PD-1 blockade in our patient is nevertheless an intriguing consideration [8].

PD-1 blockade reverts the PD-1 receptor-mediated dysfunctional state of effector memory T cells that have infiltrated the tumor into an active, functional state, thereby enabling tumor cell killing. These T cells are presumably endogenously primed and specific for antigens expressed on the respective tumor they traffic into. It therefore appears more likely that these T cells recognize tumor antigens that are distinct between the melanoma and the RCC rather than shared antigens. Although the immune response may be distinct in RCC and melanoma, a certain percentage of these tumors are immunogenic and therefore potentially responsive to immune checkpoint blockade [15]. This case illustrates that despite these distinct immune responses, PD-1 inhibition may be a suitable treatment option for patients with concurrent immune-responsive cancers.

## Consent

Clinical data for this case report were collected under institutional review board approval (Dana-Farber/Harvard Cancer Center #05-042).

### Consent to publish

Written informed consent was obtained from the patient for publication of this case report and any accompanying images.

## Abbreviations

CT: computerized tomography
CTLA-4: cytotoxic lymphocyte antigen-4
MRI: Magnetic Resonance Imaging
PD-1: immunological checkpoint programmed death
PD-L1: programmed death ligand 1
PET/CT: Positron emission tomography computerized tomography
RCC: renal cell carcinoma
TURBT: transurethral resection of a bladder tumor
VEGF: Vascular Endothelial Derived Growth Factor
